# Usutu Virus, Italy, 1996

**DOI:** 10.3201/eid1902.121191

**Published:** 2013-02

**Authors:** Herbert Weissenböck, Tamás Bakonyi, Giacomo Rossi, Paolo Mani, Norbert Nowotny

**Affiliations:** Author affiliations: University of Veterinary Medicine, Vienna, Austria (H. Weissenböck, T. Bakonyi, N. Nowotny);; Szent István University, Budapest, Hungary (T. Bakonyi); University of Camerino, Matelica, Italy (G. Rossi);; University of Pisa, Pisa, Italy (P. Mani);; Sultan Qaboos University, Muscat, Oman (N. Nowotny)

**Keywords:** Usutu virus, viruses, blackbirds, birds, bird deaths, archived tissue samples, Italy, Europe

## Abstract

Retrospective analysis of archived tissue samples from bird deaths in the Tuscany region of Italy in 1996 identified Usutu virus. Partial sequencing confirmed identity with the 2001 Vienna strain and provided evidence for a much earlier introduction of this virus into Europe than previously assumed.

In early fall 1996, an episode of wild bird deaths occurred in the provinces of Florence and Pistoia (Tuscany region), Italy. Several bird species were affected; however, most observed bird carcasses were Eurasian blackbirds (*Turdus merula*). Several animals were subjected to necropsy, which predominantly showed swollen livers and spleens, necrotizing pericloacal dermatitis, and a variety of endoparasites. Bacteriologic, virologic, and toxicologic investigations produced no conclusive results. Formalin-fixed and paraffin wax–embedded tissue samples were archived. This event was reported in an Italian veterinary journal with local distribution and thus did not receive broad attention (*1*)

Five years later in late summer 2001, similar seasonal deaths of wild birds, again predominantly blackbirds, were observed in neighboring Austria (*2*). A particular strain of Usutu virus (USUV) was determined as the causative agent of this fatal bird disease outbreak, which recurred in Austria in subsequent years (*3*), and was later identified in Hungary (*4*), Switzerland (*5*), Italy (*6*), and Germany (*7*).

There were similarities between 1996 bird deaths in Tuscany and subsequent USUV-associated bird deaths in other areas of Europe. Thus, we retrospectively analyzed archived paraffin wax–embedded material from the Tuscany cases for USUV.

## The Study

Thirty-three paraffin blocks generally containing multiple tissue samples (such as brain, liver, spleen, kidney, lung, heart, proventriculus, gizzard, intestine, pancreas, and skeletal muscle) were used for detection of USUV. Most organ samples were from blackbirds, but some were from other bird species (Table). Tissue blocks were assigned to 4 groups: group 1 (7 blocks, blackbirds found dead or severely ill during August 28–September 25, 1996); group 2 (6 blocks, blackbirds found during October 1–November 19, 1996); group 3 (10 blocks, blackbirds, starlings [*Sturnus vulgaris*], and redwings [*Turdus iliacus*] found during August 3–September 18, 1997); and group 4 (10 blocks, blackbirds and fieldfares [*T. pilaris*] found during October 5–December 20, 1997).

**Table:** Results of IHC analysis and real-time RT-PCR for Usutu virus in birds, Italy, 1996*

Group, protocol no.	Tissue	Bird species	IHC result	RT-PCR result
1				
6439-A1	Lung, kidney, spleen	*Turdus merula*	+ (kidney)	+
6439-C2	Brain	*T. merula*	+	+
6439-C3	Brain	*T. merula*	+	+
6439-C4	Brain	*T. merula*	+	+
6439-D5	Proventriculus, gizzard	*T. merula*	–	+
6439-D6	Kidney	*T. merula*	–	+
6439-D7	Liver	*T. merula*	I	+
2				
6484-A	Skeletal muscle	*T. merula*	–	–
6484-B	Spleen	*T. merula*	–	–
6484-C	Intestine	*T. merula*	–	–
6484-D	Liver	*T. merula*	–	–
6484-E	Heart	*T. merula*	–	–
6484-F	Lung	*T. merula*	I	–
3				
7665-A1	Brain	*T. merula, T. iliacus, Sturnus vulgaris*†	–	–
7665-A2	Brain	*T. merula, T. iliacus, S. vulgaris*†	–	–
7665-B1	Brain	*T. merula, T. iliacus, S. vulgaris*†	–	–
7665-B2	Brain, intestine	*T. merula, T. iliacus, S. vulgaris*†	–	–
7665-B3	Liver, lung, kidney	*T. merula, T. iliacus, S. vulgaris*†	–	–
7665-C1	Brain	*T. merula, T. iliacus, S. vulgaris*†	–	–
7665-C2	Heart	*T. merula, T. iliacus, S. vulgaris*†	–	–
7665-C3	Liver, intestine, skeletal muscle	*T. merula, T. iliacus, S. vulgaris*†	–	–
7665-D1	Brain	*T. merula, T. iliacus, S. vulgaris*†	–	–
7665-D2	Liver, kidney	*T. merula, T. iliacus, S. vulgaris*†	–	–
4				
7714–1	Heart	*T. merula, T. pilaris*†	–	–
7714–2	Liver, kidney	*T. merula, T. pilaris*†	–	–
7714–3	Spleen, bursa	*T. merula, T. pilaris*†	–	–
7714–4	Brain	*T. merula, T. pilaris*†	–	–
7714–5	Brain	*T. merula, T. pilaris*†	–	–
7714–6	Intestine, pancreas	*T. merula, T. pilaris*†	–	–
7714–7	Heart	*T. merula, T. pilaris*†	–	–
7714–8	Heart	*T. merula, T. pilaris*†	–	–
7714–9	Brain	*T. merula, T. pilaris*†	–	–
7714–10	Kidney	*T. merula, T. pilaris*†	–	–

*IHC, immunohistochemical; RT-PCR, reverse transcription PCR; +, positive; –, negative; I, inconclusive. †Blocks not assignable to individual bird species.

Recut samples from these blocks were placed on positively charged slides (Superfrost plus; Menzel Gläser, Braunschweig, Germany) and processed for immunohistochemical staining by using a rabbit USUV-specific antibody at a dilution of 1:4,000. Immunohistochemical analysis was performed by using an automated immunostainer (Autostainer 360–2D; Thermo-Fisher, Kalamazoo, MI, USA). From the same paraffin blocks, three 10 µm–thick samples were cut and used for RNA extraction.

Viral RNA was purified from paraffin-embedded tissue samples by using the QIAamp Viral RNA Mini Kit (QIAGEN, Hilden, Germany) after deparaffinization with xylene. Because of the formaldehyde fixation, the paraffin wax embedding procedure, and the long storage time, a high degree of RNA fragmentation was expected. Therefore, the PCR-based nucleic acid detection methods were specific for short (<300 nt) sequences.

In a TaqMan-based real-time reverse transcription PCR (RT-PCR), genomic (5′-GCCAATGCCCTGCACTTT-3′) and reverse (5′-TCCCGAGGAGGGTTTCCA-3′) primers amplify part of the nonstructural protein 5 (NS5) gene region of USUV between nt positions 9721 and 9795 (according to the USUV complete genome sequence, GenBank accession no. NC_006551). The TaqMan probe (FAM-5′-CGATGTCCAAGGTCAGAAAAGACGTGC-3′-TAMRA) hybridizes the amplification product between nt positions 9746 and 9773. The SuperScript III Platinum One-Step qRT-PCR System (Invitrogen, Carlsbad, CA, USA) was used for amplifications according to the manufacturer’s instructions. Primers and probe were used at concentrations of 0.2 µmol/L. Reactions were performed in an Applied Biosystems (Foster City, CA, USA) 7300 Real Time PCR System with a thermal profile of 48°C for 15 min, 95°C for 2 min, and 45 cycles at 95°C for 15 sec and 60°C, for 30 sec. Selected real-time RT-PCR virus-positive samples were also tested by using conventional RT-PCRs that generated short amplification products. Two primer pairs (Usu9247f-Usu9445r and Usu10626f-Usu10828) (*6*) amplified specific products.

Nucleotide sequences of real-time and conventional RT-PCR amplification products were determined and identified by using a BLAST search (http://blast.ncbi.nlm.nih.gov/). Sequences were aligned with USUV sequences available in GenBank. Phlyogenetic analysis with the neighbor-joining algorithm was performed to infer genetic relatedness between sequences.

**Figure 1:**
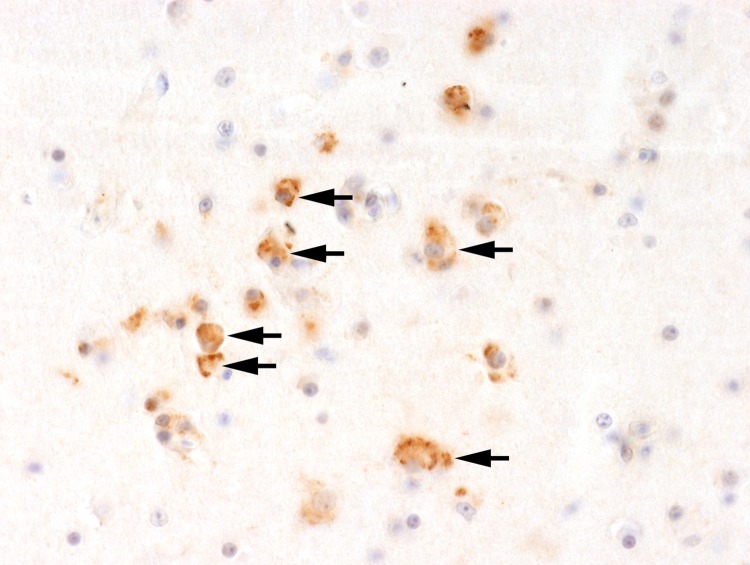
Immunohistochemical staining with Usutu virus–specific antibody showing virus antigen in the brain of a blackbird that died during an Usutu virus outbreak in Italy, 1996. Numerous neurons show characteristic, frequently coarsely granular cytoplasmic labeling (arrows). Original magnification ×390.

Immunohistochemical analysis showed positive results for USUV in several brain samples (Figure 1) and 1 kidney sample from only group 1 blocks. Liver, lung, spleen, and proventriculus were negative for USUV. In all blocks from the other 3 groups, there was no specific staining.

Results of real-time RT-PCR were positive for all samples from group 1; samples from other groups were negative. Nucleotide sequences of amplification products were 100% identical with available USUV sequences in GenBank. Selected samples (brains for group 1) were also positive by conventional RT-PCRs.

Nucleotide sequences of the amplification products in the partial NS5 gene region (between nt positions 9267 and 9425, excluding primer sequences) were 100% identical with the corresponding sequences of USUV detected in Austria in 2001 and in Italy and in Switzerland in 2006. Sequences were 98.7%–99.3% identical with other USUV sequences from Austria, Italy, Hungary, and Germany. However, these sequences were only 95.6% identical with USUV detected in Spain in 2009 and 96.2% identical with the reference strain isolated in South Africa in 1959.

**Figure 2:**
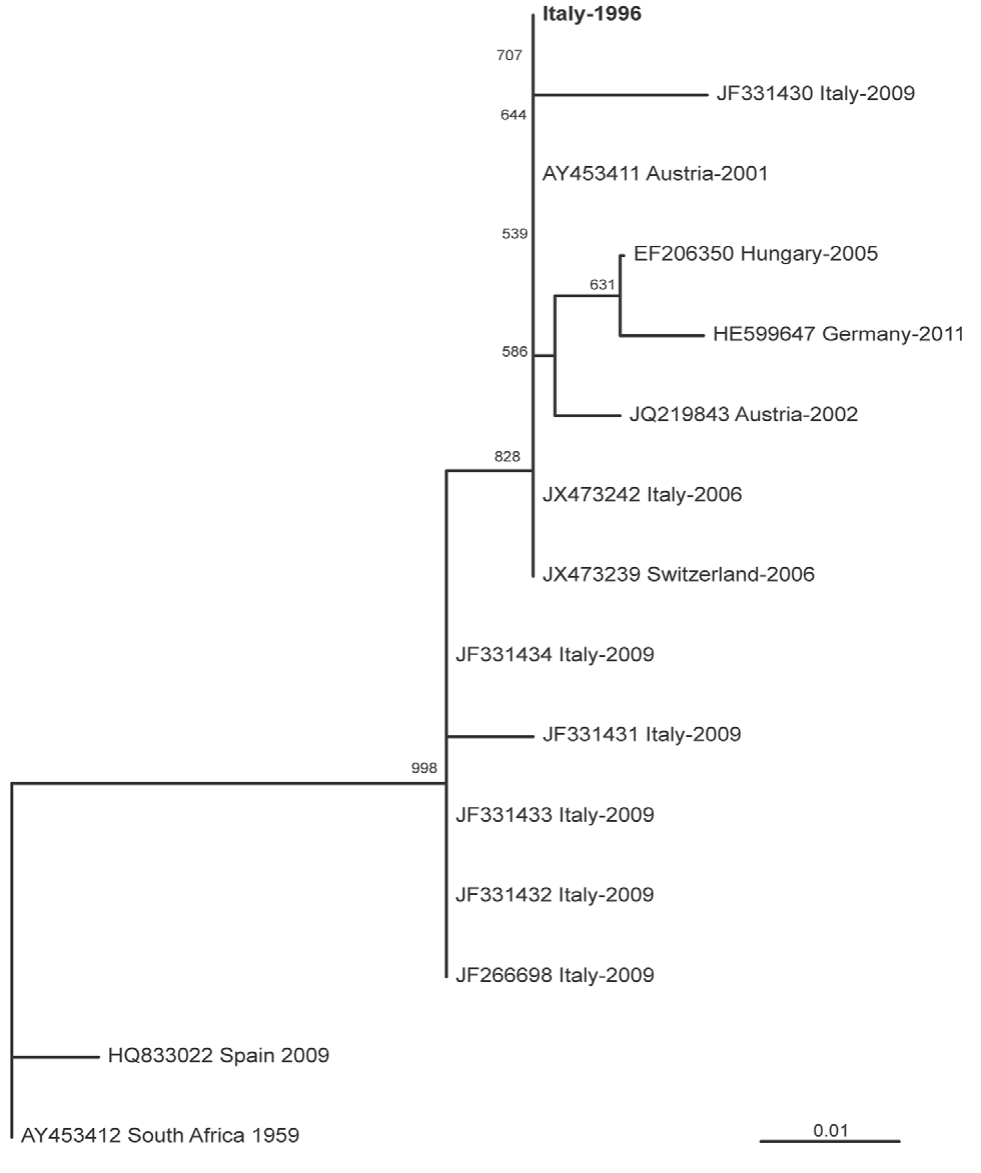
Genetic relationships of partial nonstructural protein 5 nucleotide sequences of Usutu virus, Italy, 1996. Sequences are indicated by codes containing GenBank accession number, country of origin, and year of sample collection. Virus reported in this study is indicated in **boldface**. Bootstrap values >500 (50%) are displayed. Scale bar indicates genetic distance.

Phylogenetic relatedness of sequences is shown in Figure 2. Nucleotide sequences of the 3′ untranslated region (between nt positions 10646 and 10808) were 100% identical with USUV sequences from central Europe and 98.1% identical with the reference strain from South Africa.

## Conclusions

These investigations provide evidence that USUV emerged in a pathogenic form in Europe in 1996 or even earlier. This date is >5 years before USUV-associated bird deaths in Vienna, Austria (*2*), which has been generally assumed to have been the starting point of the spread of the virus to other countries in Europe. Partial sequencing of the 1996 strain confirmed its identity with the 2001 Vienna strain and all its descendants. However, the assumed epicenter of virus spread being Austria must be reconsidered because the source has been in Italy much longer and may have given rise to subsequent local episodes of bird deaths in Italy and other countries.

USUV has an established stable mosquito-to-bird transmission cycle in Europe, which can remain silent for many seasons. There are no reports of bird deaths during 1996–2001, which might have been caused by unfavorable climatic conditions or lack of larger numbers of susceptible birds. Local herd immunity (*8*) prevented further bird deaths and supported silent spread of the virus. Large-scale wild bird deaths, as later reported in Austria, Switzerland, and Germany (*2**,**3**,**5**,**7*), had not been observed in Italy, despite widespread viral activity (*6**,**9*). Introduction of a potentially pathogenic vector-borne virus into a new area does not necessarily lead to immediate deaths, which has been repeatedly shown by seropositivity of sentinel birds or virus detection in vectors before epidemics (*10**,**11*). Episodes of bird deaths tend to occur when virus spreads to areas without prior exposure, thus affecting virus-naive birds. Also, specific climatic conditions, such as longer periods of hot and dry weather, seem to affect vector abundance and competence and efficient virus transmission to susceptible hosts (*12*).

There is evidence for introduction of other USUV strains into Europe. Direct evidence exists for a strain so far found only in mosquitoes in Spain, which is genetically different from the strain from central Europe (*13**,**14*). This strain has never been associated with bird deaths, which might have resulted from its lower virulence, but low levels of bird deaths might have occurred unnoticed. Thus, results of studies in the United Kingdom that reported several USUV seroreactive resident birds without obvious bird deaths may be explained accordingly (*15*).
